# Identification of small molecule inhibitors of ERCC1-XPF that inhibit DNA repair and potentiate cisplatin efficacy in cancer cells

**DOI:** 10.18632/oncotarget.12072

**Published:** 2016-09-16

**Authors:** Sanjeevani Arora, Joshua Heyza, Hao Zhang, Vivian Kalman-Maltese, Kristin Tillison, Ashley M. Floyd, Elaine M. Chalfin, Gerold Bepler, Steve M. Patrick

**Affiliations:** ^1^ Department of Biochemistry & Cancer Biology, University of Toledo Health Science Campus, Toledo, OH, USA; ^2^ Department of Oncology, Karmanos Cancer Institute, Wayne State University, Detroit, MI, USA

**Keywords:** ERCC1-XPF, cisplatin, chemoresistance, DNA repair inhibitor, high-throughput screen

## Abstract

ERCC1-XPF heterodimer is a 5′-3′ structure-specific endonuclease which is essential in multiple DNA repair pathways in mammalian cells. ERCC1-XPF (ERCC1-ERCC4) repairs cisplatin-DNA intrastrand adducts and interstrand crosslinks and its specific inhibition has been shown to enhance cisplatin cytotoxicity in cancer cells. In this study, we describe a high throughput screen (HTS) used to identify small molecules that inhibit the endonuclease activity of ERCC1-XPF. Primary screens identified two compounds that inhibit ERCC1-XPF activity in the nanomolar range. These compounds were validated in secondary screens against two other non-related endonucleases to ensure specificity. Results from these screens were validated using an *in vitro* gel-based nuclease assay. Electrophoretic mobility shift assays (EMSAs) further show that these compounds do not inhibit the binding of purified ERCC1-XPF to DNA. Next, in lung cancer cells these compounds potentiated cisplatin cytotoxicity and inhibited DNA repair. Structure activity relationship (SAR) studies identified related compounds for one of the original Hits, which also potentiated cisplatin cytotoxicity in cancer cells. Excitingly, dosing with NSC16168 compound potentiated cisplatin antitumor activity in a lung cancer xenograft model. Further development of ERCC1-XPF DNA repair inhibitors is expected to sensitize cancer cells to DNA damage-based chemotherapy.

## INTRODUCTION

Platinum based chemotherapy is used to treat a variety of cancers including testicular, ovarian, non-small cell lung, cervical and head and neck cancers. A major limitation is either intrinsic resistance to the chemotherapy or acquired resistance during the course of treatment. These factors limit clinical response and thus, make it important to identify factors that could increase its efficacy [1, 2].

Cisplatin interacts with DNA to form different DNA lesions that are repaired by DNA repair pathways such as Nucleotide Excision Repair (NER) [3, 4]. The ERCC1-XPF complex from the NER pathway is essential for the repair of both the intrastrand Pt-DNA adducts as well as the interstrand crosslinks (ICLs). Unrepaired lesions result in inhibition of DNA replication and transcription, which can lead to the induction of apoptosis, and ultimately, cancer cell killing and tumor regression. It has been well documented in the literature that increased DNA repair capacity of cancer cells is an important mechanism of cisplatin resistance and it is now well accepted that therapies that target DNA repair could be important in potentiating sensitivity to cisplatin and its analogues [5, 6].

Interestingly, testicular cancers that are highly responsive to platinum agents have low levels of DNA repair proteins, particularly NER proteins [7]. Another study showed that testicular tumor cell lines maintain cisplatin sensitivity due to decreased levels of ERCC1-XPF preventing ICL repair [8]. Malignant ovarian cancers that do not respond to Pt chemotherapy have increased levels of ERCC1 [9]. ERCC1 is also essential for melanoma growth and resistance to cisplatin in xenograft models. Mice with tumors that have disrupted ERCC1 survive longer on cisplatin treatment [10, 11].

We have shown that targeting ERCC1 and XPF individually or targeting the ERCC1-XPF complex can significantly enhance cytotoxicity in cancer cell lines [12]. ERCC1 has been extensively evaluated as a potential biomarker and a prognostic indicator in determining outcome as well as predicting response to Pt-based therapy [13]. Low ERCC1 mRNA levels significantly correlate with improved progression free and overall survival. Thus, ERCC1-XPF nuclease activity inhibitors hold the potential to enhance cisplatin efficacy in patients with high ERCC1 expression and potentially further increase sensitivity in patients with low ERCC1 expressing tumors.

Protein-DNA interactions have been targeted in a small number of recent studies. Small molecules have been identified for DNA damage specific DNA binding proteins, RPA and XPA, by fluorescent-based high throughput screens (HTSs) to identify small molecule inhibitors (SMIs) [14–16]. More recently small molecules have been identified that target the protein-protein interaction domain of ERCC1-XPF and increase toxicity of alkylating agents in cancer cells. Another study has also identified assays and inhibitors of ERCC1-XPF using *in silico* approaches [17, 18] while studies utilizing biochemical approaches have identified small molecule inhibitors with micromolar potency [19, 20]. More recently, the first inhibitors of the ERCC1-XPF active site and interaction domain were identified that reduced the expression of the heterodimer as well as inhibited NER activity [21]. In this current study, we describe the development of a novel fluorescence based HTS of chemical compounds to identify molecules that target ERCC1-XPF by specifically inhibiting the endonuclease activity. The endonuclease activity is specific to the ERCC1-XPF complex and compounds targeting this function would be disruptive to its DNA repair activities. Our data also indicate that the identified compounds may specifically target ERCC1-XPF's various roles in specific DNA repair pathways. Initial *in vivo* data with one of the identified compounds is extremely promising exhibiting bioavailability and potency against the tumor especially in combination with cisplatin. Finally, our screens have identified new classes of molecules with nanomolar potency against ERCC1-XPF that could be developed for therapeutic benefit in enhancing cisplatin chemotherapy.

## RESULTS

### HTS and secondary screens identify potential ERCC1-XPF inhibitors

**Figure 1:**
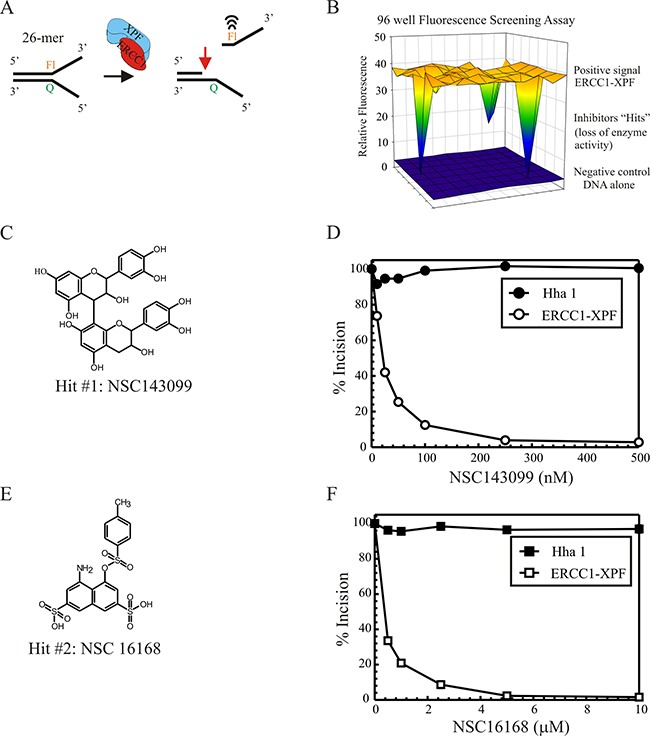
**(A)** Schematic of the DNA substrate and HTS process **(B)** 96-well plate assay for ERCC1-XPF activity. Pre-read of the plate with DNA only is indicated by the blue surface. Fluorescence measured following the addition of ERCC1-XPF is indicated by the yellow. “Hits” are indicated by the downward deflection in individual wells. **(C)** Structure of Hit #1, NSC143099. **(D)** Secondary screen of compounds from HTS testing activity against ERCC1-XPF and non-family member endonucleases, HhaI and XPG (Supplementary Figure S3A). The graph represents inhibition of fluorescence by compounds against fluorescent product from protein/DMSO control with compound titration giving specific inhibition of ERCC1-XPF and no effect on the HhaI endonuclease. **(E)** Structure of Hit #2, NSC16168. **(F)** Titration of Hit 2 to assess inhibition of ERCC1-XPF as well as HhaI. The graphs represent data from 3 individual experiments and the results are represented as the mean ± standard error.

Using the DNA substrate and the HTS assay as described in the Material and Methods we screened for the ability to inhibit the endonuclease activity of ERCC1-XPF. The NCI-DTP diversity set of ~1990 compounds was used. In the primary screens against ERCC1-XPF, 28 hits inhibited the enzyme (~1.4% initial hit rate). In secondary screens with two other non-related endonucleases (HhaI and XPG), the hits were narrowed to 12 small molecules that specifically inhibited ERCC1-XPF activity, but displayed no inhibitory effect on the other two endonucleases (~0.6% overall Hit rate). 5 of the 12 hits that were identified inhibited ERCC1-XPF enzyme activity by >90% at low μM or nM concentrations (Table 1). Figure 1A shows a typical screening assay illustrating the low background fluorescence signal of the DNA alone. When ERCC1-XPF protein was added to the reaction, a significant increase in fluorescence was observed due to the release of the fluorophore labeled incised product. The dynamic range of the positive signal with ERCC1-XPF protein above the background DNA alone and the inhibitory response observed with “Hits” in particular wells of a typical 96-well plate is shown in Figure 1B. Following the initial screening, Hits were selected based on specific activity against ERCC1-XPF and initially prioritized based on inhibition of ERCC1-XPF activity. Figure 1C shows the structure of Hit #1 (NSC143099), which has a low nM IC_50_ against ERCC1-XPF endonuclease activity (Table 1). A secondary screen was utilized to ensure specificity for ERCC1-XPF by utilizing two additional non-family member DNA endonucleases, HhaI and XPG. Titration of Hit #1 (compound NSC143099) in the HTS assay shows specific inhibition of ERCC1-XPF while no effect on HhaI activity is observed (Figure 1D). However, the compound has some effect on XPG activity at higher concentrations (Supplementary Figure S3A). Hit #2 (NSC16168; Figure 1E) also displays nM potency against ERCC1-XPF while having no effect on both HhaI (Figure 1F) and XPG (Supplementary Figure S3). Hit 1 and 2 have a very potent inhibitory activity with 50% inhibition at ~22 nM and 420 nM, respectively (Table 1; IC50s calculated by CompuSyn software and standard deviation determined by 3 different plots). Importantly, cleavage of the DNA substrate by HhaI is unaffected by these compounds and minimal to no effect on XPG cleavage demonstrating excellent specificity for ERCC1-XPF.

**Table 1:** Summary of HTS assay IC50 values

Compound Hit #:NCI #	ERCC1-XPF IC_50_ (μM)
#1: NSC143099	0.022 ± 0.003
#2: NSC16168	0.42 ± 0.112
#3: NSC103019	0.53 ± 0.128
#4: NSC14161	0.79 ± 0.35
#5: NSC13776	1.15 ± 0.96

### In vitro inhibition of ERCC1-XPF- DNA incision activity by SMIs

**Figure 2:**
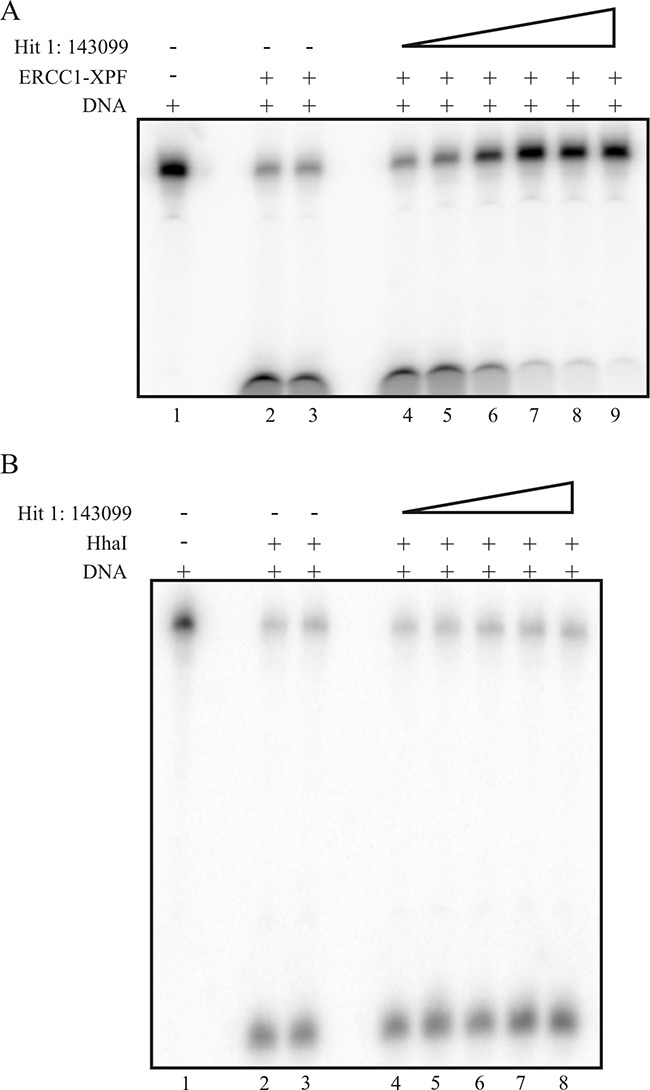
Gel based assay for ERCC1-XPF **(A)** and HhaI **(B)** activity. The 5'-[^32^P] labeled- forked DNA substrate was used which is cleaved by ERCC1-XPF and also has a HhaI recognition site. Denaturing gel electrophoresis of the products allows the separation of the substrate and products. **(A)** Gel-based assay demonstrating Hit 1 titration and inhibition of ERCC1-XPF. Lane 1 represents DNA substrate alone, lane 2 is ERCC1-XPF, lane 3 is ERCC1-XPF with vehicle control, and lane 4-9 represents Hit 1 titration, 4- 1nM, 5- 25 nM, 6- 50 nM, 7-150 nM, 8- 500 nM, and 9-1 μM. **(B)** Demonstrates compound titration and no effect on HhaI activity. Lane 1 represents DNA substrate alone, lane 2 is HhaI, lane 3 is HhaI with vehicle control, and lane 4-8 represents hit 1 titration, 4- 1 nM, 5- 25 nM, 6- 50 nM, 7-150 nM, and 8- 500 nM.

The gel-based *in vitro* incision assay has been described and extensively used [22]. Here, we titrated Hit 1 (Figure 2A) with ERCC1-XPF or control endonuclease HhaI (Figure 2B) on ice and reactions were initiated by the addition of the 5'-[^32^P] radiolabeled DNA substrate at 37°C. The products are visualized via phosphorimager analysis and the ERCC1-XPF or HhaI incised product is illustrated as a faster migrating band in the gel (Figure 2A and 2B). The data demonstrates effective inhibition of the ERCC1-XPF incision activity and correlates with our HTS data. The IC50 value from the gel-based assay for Hit 1 is ~25 nM (Figure 2A) and for Hit 2 the IC50 from the gel-based assay is ~500 nM (data not shown), very consistent with the fluorescence-based HTS assay. Next, we used the gel-based assay for compound titration with HhaI endonuclease (Figure 2B) and show that the addition of the compound does not inhibit the nuclease activity for both Hit 1 and 2 (Figure 2B and data not shown, respectively). Taken together, these data validate our HTS screening results and also demonstrate the ability to identify specific ERCC1-XPF inhibitors with low nM activity and provides an excellent platform to screen for more SMIs.

### SMIs don't inhibit the DNA binding ability of ERCC1-XPF

**Figure 3:**
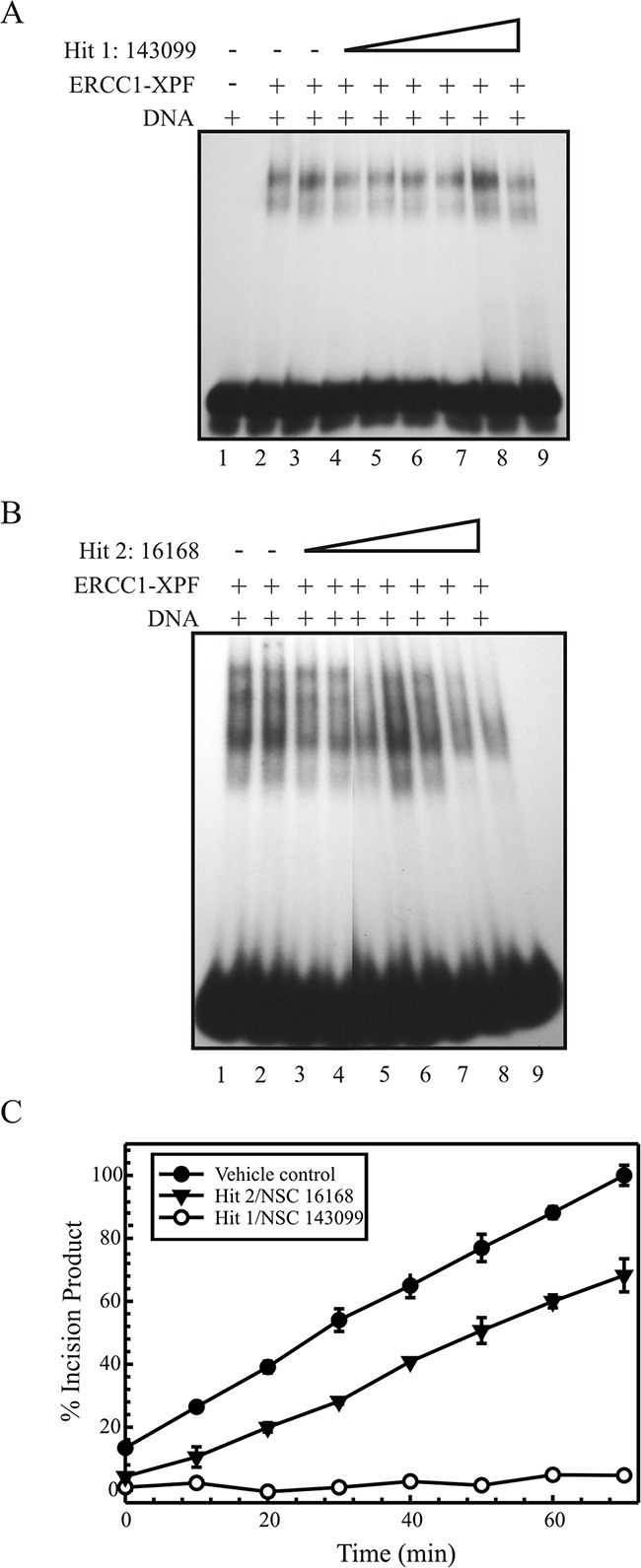
Gel based EMSA analysis for Hit 1 **(A)** and 2 **(B)**. The 5'[^32^P] labeled forked DNA substrate and ERCC1-XPF was added to the reaction buffer (buffer is without metal to prevent nuclease function) and the compound was titrated. Native gel electrophoresis allows the separation of the free DNA and that bound by ERCC1-XPF. **(A)** Lane 1 shows free DNA, lane 2 shows ERCC1-XPF bound DNA and free DNA, lane 3 with DMSO vehicle control, and lane 4-9 with Hit 1 titration, 4- 50 nM, 5- 250 nM, 6- 500 nM, 7-1 μM, 8- 15 μM, and 9-50 μM. **(B)** lane 2 with DMSO vehicle control, lanes 3 and 4 controls with 500 nM and 1 μM 143099, respectively, and lanes 5-9 with Hit 2 titration, 5- 500 nM, 6- 1 μM, 7- 10 μM, 8- 50 μM, and 9- 100 μM **(C)** Rapid dilution for enzyme and compound binding analysis. ERCC1-XPF was increased 100-fold and preincubated for 30 minutes with 10 times the IC90 of the compound or with vehicle control. This reaction is diluted to normal conditions and substrate is added and read in Spectramax M5 plate reader for 60 minutes. The values are plotted over time as % increase in fluorescent incision product or increase in fluorescent incision product over time directly correlated to ERCC1-XPF activity. Results are represented as mean ± SEM of three independent experiments. Hit 1 and 2 against vehicle control.

In order to study if the compounds' inhibitory activity of ERCC1-XPF incision is due to inhibition of DNA binding ability, we performed EMSAs [23]. One possibility for ERCC1-XPF enzyme inhibition is via inhibition of the ERCC1-XPF binding to DNA. In this assay, we incubated the titrated Hit 1 or 2 respectively with purified ERCC1-XPF on ice and reactions were initiated by the addition of the 5'-[^32^P] radiolabeled DNA substrate 37°C. The samples were separated on a 10% native gel and the products were visualized via phosphorimager analysis. Figure 3A shows the EMSA for Hit 1, wherein the titration of the compound does not affect the binding of purified ERCC1-XPF to DNA. Figure 3B shows the EMSA analysis for Hit 2, which shows similar results as Hit 1. Hit 2 at the highest concentrations appears to have a minor effect on the DNA binding but at concentrations that are used in further *in vitro* experiments, Hit 2 does not affect DNA binding of ERCC1-XPF. These data demonstrate that the ability of both Hit 1 and 2 to inhibit ERCC1-XPF endonuclease activity is not by preventing the enzyme complex from binding to DNA. In addition, studies to assess ERCC1 protein stability demonstrate that neither Hit 1 nor Hit 2 affects ERCC1 protein levels compared to cisplatin control treatment (Supplementary Figure S3B). If these compounds were affecting the protein-protein interaction between ERCC1 and XPF, we would expect to observe loss of protein following treatment similar to what we have observed with ERCC1 and XPF knockdowns [12].

Next, to determine reversible or irreversible binding, we used a rapid dilution method [24]. Here, the amount of enzyme (ERCC1-XPF) is increased 100-fold above what is normally used in a reaction and is pre-incubated for 30 minutes with a concentration of the SMI 10-times greater than the IC90 concentration or with vehicle control. After incubation, the enzyme/inhibitor mixture is diluted 100-fold (i.e., to normal reaction conditions) in the reaction buffer with the DNA substrate at normal reaction conditions. A reversible inhibitor dissociates quickly, allowing immediate recovery of enzymatic activity, whereas a slowly reversible inhibitor allows a gradual increase in activity. In contrast, an irreversible inhibitor prevents recovery of any enzymatic activity. For Hit 1, we see virtually no recovery of the enzymatic activity while Hit 2 recovers slowly (Figure 3C). These results suggest that Hit 1 binds irreversibly to ERCC1-XPF while Hit 2 appears to be a moderately reversible inhibitor.

### SMIs potentiate cisplatin cytotoxicity

**Figure 4:**
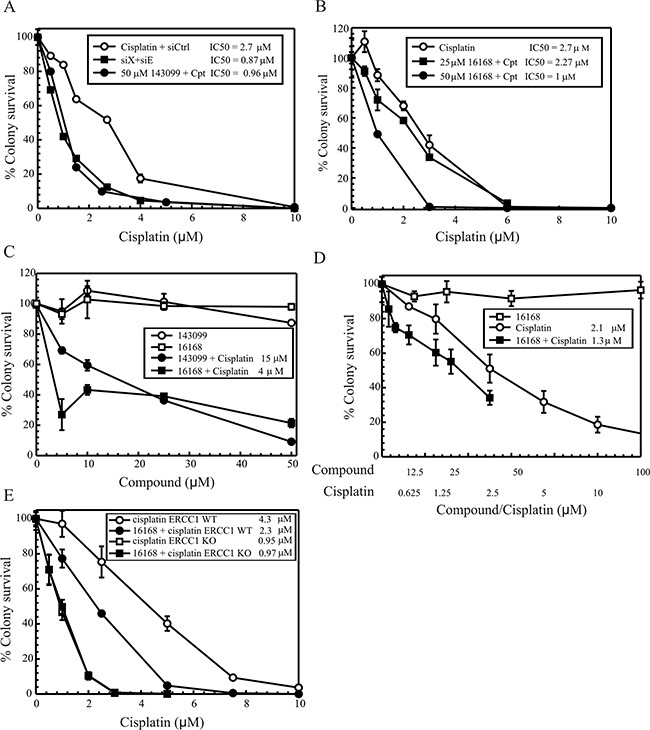
Colony survival in H460 cells (**A.** Hit 1), (**B.** Hit 2), and (**C.** Hit 1, Hit 2). In A and B, H460 cells were treated at a constant concentration of the compound and then cisplatin was titrated and treated for 2 hours. After treatment, medium was changed and cells were allowed to form colonies. **(A)** Shows comparison of clonogenic survival for non-targeting siRNA (siC, open circles) and ERCC1-XPF (filled squares denoted as siX + siE) siRNA transfected cells and Hit1 treated cells (closed circles) following cisplatin titration. Calculated IC50s are shown in the figure. **(B)** Shows clonogenic survival with cisplatin titration alone (open circles), Hit 2 at 25 μM (closed squares) and Hit 2 at 50 μM (closed circles) with cisplatin titration. **(C)** Shows titration of Hit 1/143099 or Hit 2/16168 alone (Hit 1 -open circles and Hit 2 – open squares) and Hit 1 or Hit 2 titration with cisplatin at the IC_50_ for the H460 cell line (Hit 1 - closed circles and Hit 2 – closed squares). In this graph, 100% survival has been adjusted corresponding to the cell survival at the cisplatin IC_50_ concentration. Calculated cisplatin IC50s are shown in the figure. **(D)** Titration of 16168 alone (open squares), cisplatin alone (open circles) or combination treatment (filled squares) with a constant ratio of 25:1 (16168:cisplatin) in H460 cells. The combination treatment was at the following 16168 to cisplatin concentrations (μM): 3.9:0.16, 7.8:0.32, 15.6:0.63, 31.3:1.25, 40:1.6 and 62.5:2.5. **(E)** Clonogenic survival in H1299 lung cancer cells (ERCC1 WT and KO) following 16168 incubation and cisplatin titration. Values are represented as the mean ± SEM from three independent experiments.

Our previous studies have shown that treating ERCC1-XPF knockdown cells with cisplatin significantly impacts cytotoxicity [12]. Thus for studies with the compounds, we decided to pre-treat the cells with SMIs before cisplatin treatment. We used H460, NSCLC cells, to first assess the impact on colony survival by knocking down ERCC1-XPF in these cells. ERCC1-XPF knockdown cells show a ~3 fold change in IC_50_ (Figure 4A, filled squares). Next, to assess if colony survival is affected we titrated cisplatin in these cells using a fixed concentration of Hit 1(50 μM) or Hit 2 (25 and 50 μM). As seen in Figure 4A and 4B for Hit 1 and 2 respectively, cisplatin cytotoxicity is potentiated with the compounds. The fold-change in cisplatin activity is consistent with observations following ERCC1-XPF knockdown (Figure 4A, filled squares). Next, we assessed cisplatin potentiation by treating cells at a fixed cisplatin concentration (cisplatin IC_50_ in H460 cells) with Hit 1 or 2 titration (Figure 4C, filled symbols). H460 cells were treated with compound for a total of 4 hours while cisplatin was added to the media after 2 hours of compound pre-treatment. We do not observe any additional effects if the compound containing media is left for over 24 hours (data not shown). As seen by colony survival graphs in Figure 4C, both the compounds potentiated cisplatin cytotoxicity. We also titrated Hit 1 or Hit 2 alone in these cells and treated for 4 h. Figure 4C (open symbols) shows that these compounds alone have no effect on colony survival. In Figure 4D, we treated the H460 cells with a constant ratio of Hit 2 to cisplatin at 25:1 and demonstrate a potentiation on cell cytotoxicity. We also assessed the effects of Hit 2 in another NSCLC cell line (H1299), which was either ERCC1 wildtype (WT) or knockout (KO) (Figure 4E). In the ERCC1 WT cells, Hit 2 potentiated the cisplatin response while in the ERCC1 KO cells there was no additional cisplatin potentiation, which demonstrates that Hit 2 potentiation is specific to ERCC1-XPF inhibition. Supplementary Figure S4 shows the genomic sequencing results for ERCC1 gene editing in H1299 cells and Supplementary Figure S5 is a western blot showing knockout of ERCC1 protein. Supplementary Table S1 shows sites of potential off-target editing by the ERCC1 crRNA. These potential off-targets were sequenced to confirm there was no gene editing with the ERCC1 crRNA and that knockout was specific for the ERCC1 gene.

### SMIs potentiate cisplatin cytotoxicity by targeting DNA repair

**Figure 5:**
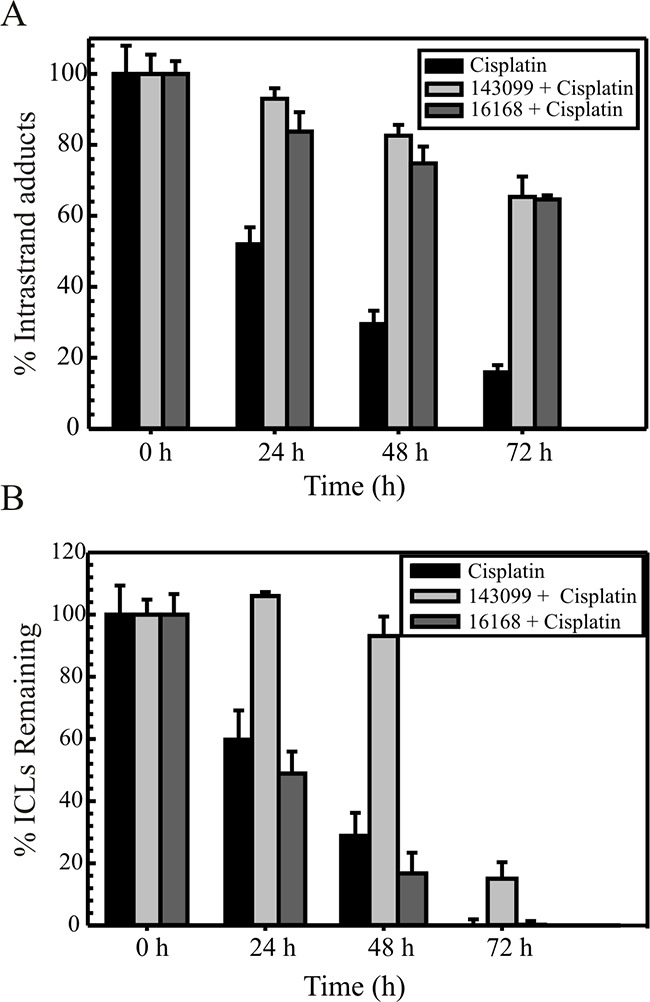
Repair of cisplatin intrastrand adducts **(A)** and interstrand crosslinks **(B)** in H460 cells. Cells were either treated with cisplatin alone for 2h at IC90 value for H460 cells or treated with Hit 1 or Hit 2 for 2 hours at 15 μM and then cisplatin at IC90 was made up in the medium and incubated for another 2 hours. **(A)** After the treatment time, genomic DNA was isolated at different time intervals (0, 24, 48 and 72 hours). ELISAs were performed as described using cisplatin intrastrand adduct antibody and the percentage of intrastrand adducts remaining was calculated at the denoted times. The results are represented as mean ± SEM of three independent experiments. **(B)** After treatment, comet assay was performed as described at different time intervals (0, 24, 48 and 72 h). The percentage of interstrand crosslinks remaining was calculated using olive tail moment. Results are represented as mean ± SEM of three independent experiments. In A and B, black bars denote cisplatin alone, light blackish-grey denotes Hit 2 and light grey denotes Hit 1.

The ability of the SMIs to inhibit the repair of cisplatin 1,2 dGpG intrastrand adducts over time was assessed by a previously described ELISA method (Figure 5A) [12]. The repair kinetics of cisplatin intrastrand adducts at various time intervals was calculated as the percent of adducts remaining over time, relative to the percent of adducts present at the 0 hour treatment (100%). In cisplatin only treated cells, the intrastrand adducts were repaired gradually from 24 to 48 hours, with ~15 % adducts remaining at 72 hours (Figure 5A). However, when we treated cells with Hit 1 or 2 (Figure 5A), the removal rate of these adducts was decreased. Hit 1 had ~65 % of adducts still remaining at the last time point tested and similarly for Hit 2 which had ~60% of adducts present at the last time point tested (Figure 5A). These data demonstrate that the compounds inhibit the repair of the major cisplatin-DNA adducts in cancer cells by likely targeting ERCC1-XPF in the NER pathway.

Next, we have previously described a modified comet assay to show that ERCC1-XPF knockdown prevents the repair of ICLs in cancer cells [12]. We show the repair kinetics of cisplatin ICLs in H460 cells with Hit 1 or 2 after 0, 24, 48 and 72 hours post treatment and compare it to treatment with cisplatin alone (Figure 5B). The data is expressed as the percentage of crosslinks remaining at the time points assessed. Cisplatin treatment induced a similar extent of ICL formation at 0 hour in cisplatin alone or when cisplatin was used in combination with either of the compounds (Figure 5B). In cells treated only with cisplatin, ICLs were removed efficiently with ~1-5 % of the ICLs remaining at 72 hours, whereas when combined with Hit 1, ICL repair was significantly reduced. At 72 hours, we see a sudden drop in repair of the ICLs, which could be attributed to the short half-life of the compound. Interestingly, with Hit 2 we see similar kinetics of ICL repair as cisplatin alone suggesting Hit 2 does not have any effect on ICL repair. This compound has been shown previously to inhibit APE1 endonuclease [25]. We have previously shown that inhibiting BER enzymes, including APE1 can result in cisplatin resistance via enhanced cisplatin ICL DNA repair [26]. It is possible that the concentrations of Hit 2 utilized for these experiments inhibit APE1 and increase the removal of cisplatin ICLs.

### *In vivo* response of Hit 2 using H460 xenografts

**Figure 6:**
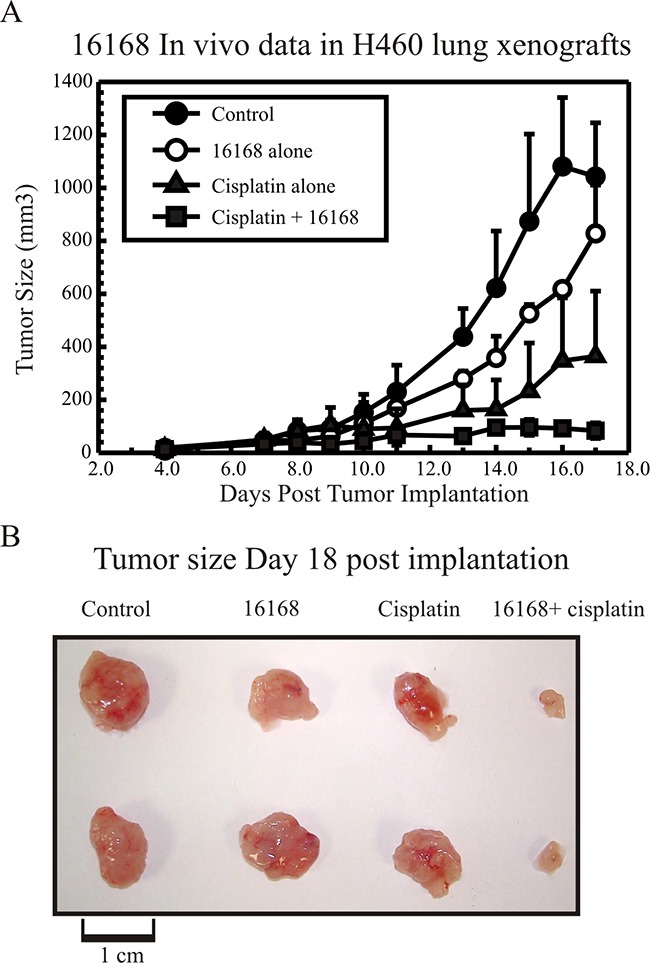
*In vivo* response of Hit 2, NSC16168 potentiating cisplatin efficacy **(A)** 2.5×10^6^ H460 cells were injected *s.c.* in the right flank of each mouse. When the tumors reached about 100 mm^3^ (day 7 after inoculation), the mice were randomly assigned into four groups: control, 16168 alone, cisplatin alone and combination of 16168 and cisplatin. Mice were treated with 16168 (20 mg/kg) *i.p.* daily and cisplatin (3 mg/kg) *i.p*. twice a week for 10 days and tumor volumes were determined as described in the “Materials and Methods” in Supplementary Information. **(B)** Tumors were harvested and pictures were taken at the end of the experiment (day 18 after inoculation). The results are the mean ± standard deviation (SD).

A pilot study was conducted to determine the toxicity and chemotherapy enhancement of Hit 2 (16168) in H460 lung cancer xenografts. In Figure 6A, vehicle control mice tumors reached an endpoint (~1000 mm^3^) in approximately 16 days. In the 16168 treated mice, the tumor growth was minimally affected by compound and the mice displayed no signs of distress or toxic side effects. In the cisplatin treated mice, there was an initial tumor response that delayed tumor growth but ultimately, the tumors continued to grow following a 2-day plateau on growth. Significantly, the combination treatment with 16168 and cisplatin inhibited H460 tumor growth, which was maintained for the duration of the compound injections. Figure 6B highlights the representative tumors harvested from mice for each group at day 18-post implantation.

## DISCUSSION

The interest in targeting DNA repair has intensified with the recent FDA approval of the first DNA repair inhibitor, olaparib, targeting poly ADP ribose polymerase (PARP) [27]. Excitingly, preclinical and clinical data indicate that inhibition of ERCC1-XPF could modulate the repair of cisplatin-induced DNA damage and impact patient response to platinum based therapies [28–31].

In this study, we describe a fluorescent-based screen to identify compounds that specifically inhibit ERCC1-XPF. From the primary and secondary screens with the HTS assay, we selected the top two Hits that inhibited ERCC1-XPF in the nanomolar range. These hits were further validated *in vitro* using a gel based nuclease incision assay. Inhibition values from both the HTS assay and the gel assay were the same for both compounds (Table 1). Secondary screens with two non-related endonucleases further validate specificity of these two hits to ERCC1-XPF. While XPF has the nuclease domain, ERCC1 has the central domain that binds the complex to DNA and the DNA binding ability is essential for the function of the complex [31]. EMSA results for DNA binding activity show that these hits do not inhibit the binding of ERCC1-XPF to DNA at concentrations that are effective at inhibiting the enzymatic activity (Figure 3). In addition, these compounds do not alter the stability of either ERCC1 or XPF, which strongly suggests that these compounds do not disrupt the protein-protein interaction (Supplementary Figure S3B). These data suggest that these compounds are potentially binding either to the active site of ERCC1-XPF or allosterically inhibit the endonuclease function. We studied the reversibility or irreversibility of the interaction between the compound and ERCC1-XPF by the rapid dilution method [24]. We identified Hit 1 as an irreversible inhibitor of ERCC1-XPF while Hit 2 is slowly/moderately reversible. Future lead compound development could employ other sensitive methods to delineate these inhibitory mechanisms.

To study the effect on cisplatin efficacy, we tested the two Hits in H460 and H1299 lung cancer cells, where the Hits alone do not show any cytotoxic effects (Figure 4C and 4D). However, in combination with cisplatin, we observed a ~3-fold change in cisplatin IC_50_ with Hit 1 and 2 (Figure 4). DNA repair assays measuring the repair of cisplatin-DNA lesions correlated well with the decreased colony survival (Figure 5). Intrastrand adducts are the major cisplatin-DNA lesion (65-85%) and are normally repaired by NER, where ERCC1-XPF plays a central role as an endonuclease [12]. Compounds that target the repair of these lesions could also potentially be used for other agents that utilize the NER pathway for repair. We used a modified comet assay to study the repair of cisplatin ICLs following compound treatment (Figure 5B). We show that Hit 1 is a good inhibitor of ICL DNA repair. Interestingly, Hit 2 does not appear to inhibit ICL repair and has similar repair kinetics as cisplatin alone. This could mean that Hit 2 may inhibit a specific region of the enzyme that is responsible for protein or enzymatic interactions required in the NER pathway. In this scenario, Hit 2 would not inhibit specific ERCC1-XPF activities required for ICL DNA repair. Interestingly, Hit 2 (NSC16168) has been shown to have some activity inhibiting the APE1 endonuclease (IC50 = ~7 μM) required for base excision repair (BER) [25]. In our previous work, we have demonstrated that targeting BER factors including APE1, results in a cisplatin resistant phenotype via enhanced ICL DNA repair [26]. It is possible that what we observe in the ICL repair assay is in fact due to Hit 2 inhibition of APE1, which could alter the cisplatin ICL DNA repair independent of the inhibition of ERCC1-XPF. It is also possible that due to the moderately reversible nature of this inhibitor, we see differential responses to the various DNA repair pathways. Further testing is needed to address these possibilities.

SAR studies with stereoisomers of Hit 1 (NSC143099, Figure 1C) show that, stereochemistry plays an important role in the activity of the compound towards inhibition of ERCC1-XPF. We also tested these different isomers in cell culture and found activity for only one of the structural analogues (Procyanidin B3, Supplementary Figure S6). Unfortunately, we were unable to test all the known stereoisomers due to limited commercial availability. Core structures or half structures of Hit 1 that we tested showed minimal activity in the assays. Additional studies assessing structural analogues will be important for addressing specific chemical groups critical for mediating the inhibition of ERCC1-XPF and would be critical for future “drug-like” compound development. Although Hit 1 (NSC 143099, Figure 1C) is a good inhibitor *in vitro*, it has a relatively high molecular weight (578 g/mol) and possesses more than 5 hydrogen bond donors and acceptors. These properties violate Lipinski's rules for proper drug-like activity, thus making it an unsuitable drug candidate.

Hit 2 (NSC16168, Figure 1E), has a molecular weight of 473 g/mol and follows Lipinski's rules and has phenyl and biphenyl hydrophobic groups that help in absorption. However, sulfonic acid esters could potentially be toxic due to alkylation. These groups can also hinder absorption, thus if these groups can be replaced it could enhance the potential of this compound as a lead molecule. More importantly, in pilot mouse xenograft studies, Hit 2 showed no toxic effects alone in mice and in combination with cisplatin showed a significant effect on tumor growth compared with cisplatin alone (Figure 6). We screened commercially available core structures of Hit 2 or with sulphonic substitutions. The results from these studies are also summarized in Supplementary Figure S6. The core structure of Hit 2 with sulfonic acids retains activity against ERCC1-XPF incision (~3.5 mM IC_50_), but it does not potentiate cisplatin activity in colony survival experiments. We will further test commercially available structural analogues for both Hit 1 and 2 in future SAR studies especially in light of the new *in vivo* evidence. In addition, medicinal chemistry efforts will be utilized to synthesize analogues of NSC16168 which shows great promise in mouse xenograft studies in combination with cisplatin. Larger scale screening efforts using better drug-like libraries will also provide a structurally diverse set of compounds that can be further developed for better potency.

In conclusion, we have developed a novel fluorescent HTS to identify compounds that target ERCC1-XPF endonuclease activity and decrease DNA repair function, which ultimately enhance cisplatin sensitivity. Unfortunately, Hit 1 (NSC143099) had a high affinity to purified enzyme but lacked potency in cell culture studies (IC50 ~25 nM versus purified enzyme and IC50 ~15 μM to impact cisplatin efficacy). There are a variety of possible reasons for this lack of cellular activity for Hit 1 including off-target protein binding of the compound, inactivation of compound or cellular uptake issues to name a few. Importantly, our studies identify a compound (Hit 2, NSC16168) that not only targets purified ERCC1-XPF but also potentiates cisplatin efficacy in cell culture experiments. In addition, the pilot *in vivo* xenograft data (Figure 6) using H460 lung cancer cells provide the proof of principle that targeting ERCC1-XPF can enhance cisplatin potency and also highlights NSC16168 as a potential lead structure for future drug development efforts towards targeting ERCC1-XPF.

## MATERIALS AND METHODS

### Design and methodology of the HTS fluorescence assay

To screen for ERCC1-XPF inhibition, an existing *in vitro* assay was optimized to a 96-well plate format to allow for screening of compound libraries (NCI-DTP diversity set). These compounds and subsequent vialed compounds were from the Drug Synthesis and Chemistry Branch, Developmental Therapeutics Program, Division of Cancer Treatment and Diagnosis at the National Cancer Institute. We used purified ERCC1-XPF (Supplementary Figure S1) and describe the purification procedure in Supplementary Information. The initial cuvette-based assay was optimized for DNA, MgCl_2_ concentrations as well as the incubation time to be used in the fluorescence assay in a 96 well plate format (Supplementary Figure S2). The assay consists of a 10 nM DNA (Q+Fl substrate annealed, described below) and 7.5 nM ERCC1-XPF protein in buffer containing 50 mM Tris-HCl pH8.0, 2 mM MgCl_2_, 0.1 mM BSA, and 0.5 mM β-mercaptoethanol. For preliminary screening, 150 μL of DNA was added to each well and the fluorescence signal was measured at 525 nm following excitation at 485 nm. Compounds or controls were then added to individual wells for a total of 80 compounds on each plate and the fluorescent signal was re-measured. We initially ran the screen using 50 μM compound and then repeated using 10 μM compound. 16 controls were used per plate, which consisted of buffer alone, DNA alone, ERCC1-XPF alone, 10 nM fluorescein ssDNA (positive signal), and 10 nM Q-Fl DNA with 7.5 nM ERCC1-XPF (positive signal). 7.5 nM ERCC1-XPF was added to each well containing the compounds and DNA and incubated for 30 minutes at 37°C after which the fluorescent signal was measured again.

The synthetic DNA substrate mimics a native forked ssDNA-dsDNA substrate of ERCC1-XPF (Figure 1A). This forked DNA substrate has a dsDNA region (14 bases) and a region containing two ssDNA flaps (12 bases each). One 26-mer DNA oligonucleotide contains a site-specific fluorescein modification at the indicated position (Fl Oligo: 5' - GCCAGCGCTCGGAT(AminoC6dT)(FLSN) TTTTTTTTTTT). The semi-complementary strand was synthesized containing a DABCYL quencher (Q) molecule at the depicted position (Q Oligo: 5' - TTTTTTTTTTT (AminoC6dT)(Dabcyl) ATCCGAGCGCTGGC; Figure 1A). The fluorescein (*) molecule has an excitation peak centered at 485 nm and an emission peak centered at 525 nm. The quencher (Q) molecule is able to quench the fluorescence signal of the fluorescein when it is in close proximity. The design of the semi-duplex DNA substrate is such that the fluorescein and DABCYL quencher are directly opposite one another at the double-stranded and single-stranded DNA junction (Figure 1A). This results in a significantly quenched signal when excited at 485 nm measuring emission at 525 nm. Upon cleavage by ERCC1-XPF, (~4-5 bases from the dsDNA-ssDNA) the fluorescein label on the cleaved DNA is released into solution and results in a significant increase in fluorescence as the quencher is no longer in close proximity. The DNA substrate is also incorporated with a restriction enzyme site (HhaI) within the duplex DNA that results in a cleavage pattern similar to ERCC1-XPF. This serves as a positive control and allows assessment of ERCC1-XPF cleavage product. This DNA substrate is used in the solution based fluorescence screening assay to screen for inhibitors of ERCC1-XPF endonuclease activity. In the 96 well platform, the Z factor was calculated to be ~0.87 indicating a highly robust assay and highly suitable for a HTS. A Molecular Devices Spectramax M5 plate reader was used for fluorescence detection. The DNA substrate was the same for gel-based assays besides the fluorescent/quencher modifications and 5'-labeling using γ-ATP^32^. For the secondary screen, the reaction was setup in a tube with 10 nM DNA, 7.5 nM XPF-ERCC1, 20 U/ml of HhaI, or 7.5 nM XPG (Supplementary Figure S3A) in nuclease reaction buffer with compound titration and incubated at 37°C for 30 minutes. The reaction was stopped by chelating the metal in the buffer and read in the Spectramax M5 plate reader.

### “Hit” validation in secondary gel-based assay

Hits from the HTS fluorescence screen were validated in a gel based nuclease activity assay as previously described [22]. This assay is robust and highly quantitative. Briefly, the 26- mer DNA substrate (Figure 1) was labeled on the 5'-terminus with [γ^32^P]-ATP using T4 polynucleotide kinase (NEB) for 30 minutes at 37°C. The 5'-labeled DNA substrate was annealed to its complementary strand by heating to 95°C for 5 minutes, followed by 65°C and then 37°C for a total of 2 hours. This substrate was gel-purified on a 10% native polyacrylamide gel in TBE (Tris, borate, EDTA electrophoresis buffer), developed, cut and kept for gel elution overnight at 4°C. Post gel elution, the substrate was ethanol precipitated and the counts were determined. The final substrate was stored at −20°C. Reactions were carried out in a volume of 8 μl at 37°C for 30 minutes in reaction buffer containing 50 mM Tris pH 8, 0.5 mM β-mercaptoethanol, 0.1 mg/ml bovine serum albumin (BSA) and 0.75 mM MgCl_2_. 10 femtomoles of the DNA substrate was added to the reaction with 15 femtomoles of the purified ERCC1-XPF enzyme or 20 U/ml of HhaI and the compound was titrated in a 8 μl reaction. The reaction was stopped by adding formamide/EDTA and the samples were heated for 5 minutes at 95°C before gel loading. Incision products were separated on a 12% sequencing gel for 2 hours. The gel was removed, dried and products were visualized by autoradiography, or on a STORM phosphorimager (Molecular Dynamics). For each experiment we used the following controls: DNA alone, DNA with ERCC1-XPF and with vehicle control.

## SUPPLEMENTARY FIGURES AND TABLE



## Abbreviations

NER: nucleotide excision repair
NSCLC: non-small cell lung cancer
ICL: interstrand crosslinks
DSBs: double strand breaks
ERCC1: excision repair cross- complementation group 1
XPF: xeroderma pigmentation group F (ERCC4)
siRNA: small interfering RNA
StaRT–PCR: standardized reverse transcription–polymerase chain reaction
ssDNA: single-stranded DNA
dsDNA: double-stranded DNA
ACTB: β- actin
ELISA: enzyme linked immuno-absorbent assay
SAR: Structure activity relationship
HTS: High throughput screen
Pt: Platinum
SMI: small molecule inhibitor
EMSA: electrophoretic mobility shift assay
WT: wildtype
KO: knockout
SEM: standard error of the mean
s.c.: subcutaneous
i.p.: intraperitoneal.
