# Supplementary material for: Identification of small molecule inhibitors of ERCC1-XPF that inhibit DNA repair and potentiate cisplatin efficacy in cancer cells
Source: Oncotarget. 2016 Sep 16;7(46):75104–17. doi: 10.18632/oncotarget.12072 (PMC5342726; doi:10.18632/oncotarget.12072)
Supplement: Supplementary file 1 [file oncotarget-07-75104-s001.pdf]

Identification of small molecule inhibitors of ERCC1-XPF that inhibit DNA repair and potentiate cisplatin efficacy in cancer cells

SUPPLEMENTARY INFORMATION

MATERIALS AND METHODS

Chemicals

Cisplatin [cis-diammine-dichloroplatinum (II)] was purchased from Sigma –Aldrich. The antibodies were polyclonal ERCC1 Fl-297 (sc-10798, Santa Cruz) and monoclonal XPF (MS-1381-PIABX, Neomarker). We used the Qiagen DNeasy blood and tissue kit for DNA isolation and Profoldin DNA binding plates for the ELISA. ICR4 antibody was kindly provided by Michael J. Tilby, University of New Castle, UK. All other reagents and chemicals were from standard suppliers. Oligonucleotides were purchased from Midland DNA technologies and HPLC and gel purified to ensure that the DNA contains the appropriate modification. The NCI-DTP diversity set was used for screening. Hit 1 and 2 were identified from the NCI-DTP diversity set screen.

Cell culture

NSCLC cell lines, H460 (provided by Dr. James C. Willey, University of Toledo) and H1299 (provided by Dr. Gerold Bepler) were maintained in RPM1 1640 supplemented with 10 % FBS in the presence of penicillin (100 IU/ml) and streptomycin (100 µg/ml). Cells were grown at 37 °C in a 5 % CO_2 incubator. These cell lines were authenticated by the Biobanking and Correlative Sciences Core at Karmanos Cancer Institute using standard procedures.

ERCC1-XPF protein purification

The ERCC1 and XPF cDNA were co-overexpressed in SF9 insect cells using a baculovirus expression system (obtained from Aziz Sancar, University of North Carolina) as previously described [1]. Briefly, 48 hours post-infection virus expression, cells were collected, washed and cell free extracts were prepared as described previously. Initial fractionation was performed on a 40 ml phosphocellulose column, which was initially loaded and washed with a low salt buffer. Bound protein fraction containing ERCC1-XPF was eluted in high salt (500mM NaCl) and supplemented with imidazole to 10 mM. The eluted protein was directly applied to a 5 ml Ni-NTA agarose column and washed with buffer containing 50 mM imidazole. ERCC1-XPF was eluted with 250 mM imidazole and active fractions were determined via nuclease activity using a fluorescence assay as described in the methods section for HTS fluorescence assay and fractions with greater than 50 % maximum eluted activity were pooled and dialyzed in low salt buffer. Further fractionation was performed by S-Sepharose and Q-Sepharose chromatography to make sure all nuclease activity was a function of ERCC1-XPF catalyzed cleavage. The final pool of protein was stored at -80°C. The highly purified ERCC1-XPF is very stable and displays no loss of activity over 6 months. Typical yields are 0.5-1 mg/L of infected Sf9 insect cells. Supplemental Figure S1A shows the different fractions from the purification process and the highly purified ERCC1-XPF enzyme that had nuclease activity and was free from non-specific nuclease activity (Supplementary Figure S1B).

Fluorescent DNA incision reaction

For the assessment of activity, a 48 base DNA substrate was utilized that forms a ‘loop’ structure. The oligo sequence was: 5’- GCCAGCGCTCGGATTTT TTTTTTTTTTTTTTTTTTACCGAGCGCTGGC-3’ Experiments for the cuvette reactions were performed in reaction buffer containing 50 mM Tris, pH 8.0, 0.5 mM $MnCl_2$, 0.1 mg/ml BSA, and 0.5 mM β-mercaptoethanol in a final volume of 500 µl.

DNA Binding-electrophoretic mobility shift assay (EMSA)

The labeled DNA substrate (as described above) was used for testing the effect of compounds on the DNA binding activity of ERCC1-XPF to DNA. As ERCC1-XPF requires metal for DNA incision, $MgCl_2$ was excluded from the reaction buffer. 0.1M EDTA was added to the reaction buffer and the reactions were carried out in a similar manner as described for the gel-based incision assay. Post incubation, glutaraldehyde was added to a final concentration of 0.25% and samples were incubated for 5 minutes at 37°C. The reaction was run on a 10% native polyacrylamide gel in TBE at 4°C (Tris, borate, EDTA electrophoresis buffer). The gel was removed, dried and products were visualized by autoradiography, or on a STORM phosphorimager (Molecular Dynamics). For each experiment, we used the following controls: DNA alone, DNA with ERCC1-XPF and/or with vehicle control. Hit 1 or Hit 2 were titrated into the reaction as follows: Hit 1

(NSC143099) was titrated 10 nM, 50 nM, 250 nM, 500 nM, 1 µM, 15 µM and 50 µM. Hit 2 (NSC16168) was titrated at 500 nM, 1 µM, 10 µM, 50 µM and 100 µM.

Analysis of DNA binding

Rapid dilution experiments as described previously [2] were used to demonstrate reversible or irreversible binding of the identified compounds – Hit 1 (NSC143099) and Hit 2 (NSC16168) to ERCC1-XPF. Briefly, in a 10 µl reaction, ERCC1-XPF concentration was increased 100-fold from normal reaction conditions of 7.5 nM and mixed with 10 times the IC90 concentration of the compound or vehicle control (9:1 DMSO-glycerol). After incubation at 37°C for 30 minutes, 2 µL of the pre-incubate was diluted into a 198 µL solution containing the DNA substrate and reaction buffer described in the methods section for HTS assay in a 96 well plate. The 96-well plate was read in a Spectramax M5 repeatedly for 60 minutes. The data was plotted as increase in the fluorescent incision product showing the activity of ERCC1-XPF against the DNA substrate.

Cisplatin intrastrand adduct measurement by ELISA

Repair of intrastrand adducts was assessed by ELISA as described with some modifications [3]. Cells were treated with compound (Hit 1 or Hit 2) and cisplatin or just cisplatin alone in serum free media. For treatment with compound and cisplatin, cells were treated with compound at 15 µM for 2 hours and then cisplatin was added (at IC90 concentration for the cell line used) to the media and these were incubated for another 2 hours. Cells were then washed with PBS and fresh medium was added. At various time points between 0 and 72 hours after drug treatment, genomic DNA was isolated and sonicated for 30 – 60 seconds in Cole Palmer ultrasonic processor. Equal amounts of DNA were coated on 96 well DNA binding ELISA plates in binding buffer (1M sodium chloride, 50 mM sodium phosphate buffer, pH 7.4, 0.02% sodium azide) and incubated at 4°C overnight. The wells were blocked with 1% BSA in PBS for 1 hour at room temperature. ICR4 antibody diluted 1:2000 in dilution buffer (0.2% BSA, 90 mM sodium chloride, 0.2% Tween-20 in PBS) was added to the wells and incubated at 37ºC for 1 hour. Following three washes with washing buffer (0.1% Tween-20 in PBS), HRP conjugated goat anti-rat antibody diluted 1:2500 (1% BSA, 0.2% Tween-20 in PBS) was added to the wells and incubated at 37ºC for 30- 60 minutes. After five washes with washing buffer, TMB (1 step ultra TMB-ELISA, Thermo Scientific) was added and kinetics of absorbance was measured at 650 nm in a Spectramax M5 plate reader (Molecular Devices) for 15 minutes. The reaction was stopped by adding 1M sulfuric acid and absorbance was measured at 450 nm. All samples were assayed in triplicates. The mean background (antibody blank) was subtracted from all the readings and the % intrastrand adducts were calculated using OD 450 nm where the 0 hour time point (2 hours post cisplatin treatment) was used as 100% intrastrand adducts in each cell line.

Cisplatin interstrand crosslink measurement by comet assay

Repair of interstrand crosslinks (ICLs) was assessed by alkaline comet assay with some modifications [3]. Cells were treated with compound (Hit 1 or Hit 2) and cisplatin or just cisplatin alone in serum free media. For treatment with compound and cisplatin, cells were treated with compound at 15 µM for 2 hours and then cisplatin was added (at IC90 concentration for the cell line used) to the media and these were incubated for another 2 hours. At the end of treatment, cells were washed with PBS and incubated in fresh medium for the required post-incubation time or assayed immediately (time 0 hour). Cells were further treated with 100 µM of hydrogen peroxide for 15 minutes to induce random DNA strand breaks. Cells were then trypsinized, pelleted and resuspended in 1% low melting point agarose. Cell samples (~10,000 cells) were embedded on a microscopic slide precoated with 1% normal melting point agarose. Another layer of 0.5% low melting point agarose was added and allowed to solidify. The slides were then incubated in lysis solution (2.5 M NaCl, 10 mM Tris, 100 mM EDTA, pH 10, containing 1% v/v Triton X-100) for 1 hour at 4ºC in the dark. The slides were then transferred to an electrophoresis tank containing ice-cold alkaline solution (300 mM NaOH, 1 mM EDTA, pH > 13), incubated for 20 minutes to allow DNA unwinding to occur and electrophoresis was carried out for 30 minutes at 0.7 v/cm, 300 mA. Slides were removed and kept in neutralizing solution (0.4 M Tris–HCl, pH 7.5) for 10 minutes. Slides were then stained with SYBR green (Trevigen) and comets were analyzed using a Nikon epifluorescence microscope at 200x magnification. Fifty cells were analyzed per slide using Komet Assay Software 5.5F (Kinetic Imaging, Liverpool, UK). The degree of DNA interstrand cross-linking present in cisplatin-treated sample was determined as described by comparing the tail moment of cisplatin + H_2O_2 treated samples with H_2O_2 treated samples and untreated control samples. The level of interstrand cross-linking was calculated by the following formula: $[1 – (TM_{pt} – TM_{ctl})/(TM_{H2O2} – TM_{ctl})] \times 100$, where TM_{pt} is the mean tail moment of the cisplatin + H_2O_2 treated sample, TM_{ctl} is the mean tail moment of the untreated control sample and TM_{H2O2} is the mean tail moment of H_2O_2 treated sample and was expressed as the percent of ICLs that remained at that particular time point.

Colony survival assay

Cells were split and seeded at a density of 300-400 cells in a 60 mm plate and incubated overnight. The next day, the cells were treated with a fixed concentration of the compound (Hit 1 or Hit 2) with cisplatin titration. After treatment, fresh complete medium with antibiotics was added and the cells were then allowed to form colonies. In another method, the cells were seeded onto 60mm plates and the next day, compound (Hit 1 or Hit 2) was titrated alone or titrated with a fixed concentration of cisplatin (IC50 for the cell line used). Post treatment, the cells were allowed to form colonies. Colonies were fixed with 95% methanol and stained with 0.2 % crystal violet. Colonies with ≥50 cells were counted using a light microscope. Cell survival was expressed as the ratio of average number of colonies in drug treated cells versus control cells x 100. The experiment was done in triplicate for each drug concentration. We have previously published the methods for ERCC1 and XPF knockdowns, which were used as controls [3]. Briefly, cells were seeded onto six-well plates (density 2.5×10^5 cells /well) in antibiotic free media. Two transfections were done at 24 h interval in each cell line to knockdown ERCC1-XPF together according to the manufacturer's (Dharmacon's smartpools) protocol. Addition of lipid reagent (Dharmafect 2, Dharmacon) without the siRNA is described as mock transfection.

ERCC1 knockout with CRISPR-Cas9

Cas9-lentivirus was produced using the Lenticrispr V2, pVSVg, and psPAX2 plasmids (Addgene) in HEK293T cells as previously described [4]. The day before lentiviral transduction, one million H1299 lung cancer cells were seeded. The following day, cells were transduced for ~16-hours with Cas9 lentivirus. Cas9 expression was driven by an EFS-NS promoter. Cells were selected with 0.8 µg/mL puromycin and clones were selected using standard methods. Cas9 expression in selected clones was assessed by Western Blot and a high-expressing Cas9 clone was chosen for knockout experiments. The CRISPR RNA (crRNA) was designed using the Optimized CRISPR Design tool (crispr.mit.edu) and a crRNA targeted to the sense-strand within exon 2, the first coding exon, of ERCC1 was selected with the sequence 5' AGGGACCTCATCCTCGTCGAGGG 3'. Synthetic tracrRNA and crRNA was purchased from Dharmacon. Transfection was performed as per the manufacturer protocol. The day before transfection 300,000 cells were seeded in antibiotic-free RPMI media. The following day synthetic RNA was diluted to a 100µM stock in a 10mM Tris pH 7.4 buffer containing nuclease-free water. A final concentration of 50 nM was used for both tracrRNA and crRNA and was transfected with 3µg/mL Dharmafect Duo Transfection Reagent (Dharmacon) in a total reaction volume of 2.4 mL in a six-well plate format. Cells were transfected for 48 hours after which complete media was added for 24 hours. Cells were seeded for clones and clones were selected using standard methods. ERCC1 KO clones were tested for ERCC1 and XPF expression by Western Blot using the ERCC1 antibodies, 8F1, FL-297, and D-10 and the XPF antibody 3F2/3 (Santa Cruz). A clone with no detectable ERCC1 was selected. Validation of genome editing was performed by PCR amplification with Taq polymerase (NEB) of exon 2 within the genomic DNA using the forward primer: 5' TCGCTCCTGGCACCTTCCCTTTGAGGC 3' and the reverse primer: 5' CCAAGGACTGTT TCCCCAAGTCGTCCG 3'. PCR product was cloned into a linearized pCR4-TOPO vector and transformed into OneShot TOP10 *E. coli* using the TOPO-TA Cloning Kit for Sequencing (Thermofisher). Bacterial colonies were selected with ampicillin and plasmid was extracted using standard procedures. Plasmid was sequenced by GeneWiz using an M13R primer. Potential sites of off-target editing in coding sequences were identified during guide RNA design with the Optimized CRISPR Design Tool. Primers were designed to amplify the exons containing the potential off-target sites and PCR product was sequenced by GeneWiz. A list of specific off-target sites and primers used to assess these off-target effects are available in Supplementary Table S1. No off-target editing was identified in any of the sequenced sites.

In vivo response of Hit 2 (16168) using H460 xenografts

Female Athymic nude mice aged 5 weeks (Envigo RMS, Indianapolis, IN) were maintained in accordance with protocols approved by the Institutional Laboratory Animal Care and Use Committee of Wayne State University. H460 lung cancer cells (2.5×10^6) were suspended in 0.1 mL serum-free and antibiotic-free RPMI medium and inoculated subcutaneously (*s.c.*) in the right flank of each mouse. When the tumors reached about 100 mm^3 (on day 7 after inoculation), the mice were randomly assigned into four groups: control (6 mice), 16168 alone (3 mice), cisplatin alone (6 mice) and combination of 16168 and cisplatin (5 mice). The control group mice received a daily intraperitoneal injection (*i.p.*) injection with 100 µl of vehicle [20% DMSO, 80% Cremophor:ethanol (3:1)] while the mice for 16168 alone and combo treatment group received 16168 (20mg/kg) in 100 µl of the vehicle daily. After one day treatment of 16168 (day 8 post inoculation), the mice for cisplatin alone and combo treatment received *i.p.* injection of cisplatin (3 mg/kg) in 100 µl of saline twice a week. Tumor sizes were measured daily using calipers and their volumes calculated using a standard formula: $width^2 \times length/2$. The mice were sacrificed after 10 day-treatment of 16168 and 4 doses of

cisplatin (day 18 after inoculation) when control tumors reached about 1000 mm^3.

REFERENCES

1. Park CH, Bessho T, Matsunaga T and Sancar A. Purification and Characterization of the Xpf-Ercc1 Complex of Human DNA-Repair Excision Nuclease. J Biol Chem. 1995; 270:22657-22660.
2. Liu TC, Toriyabe Y, Kazak M and Berkman CE. Pseudoirreversible Inhibition of Prostate-Specific Membrane Antigen by Phosphoramidate Peptidomimetics. Biochemistry-Us. 2008; 47:12658-12660.
3. Arora S, Kothandapani A, Tillison K, Kalman-Maltese V and Patrick SM. Downregulation of XPF-ERCC1 enhances cisplatin efficacy in cancer cells. DNA Repair. 2010; 9:745-753.
4. Ran FA, Hsu PD, Wright J, Agarwala V, Scott DA and Zhang F. Genome engineering using the CRISPR-Cas9 system. Nat Protoc. 2013; 8:2281-2308.

SUPPLEMENTARY FIGURES AND TABLE

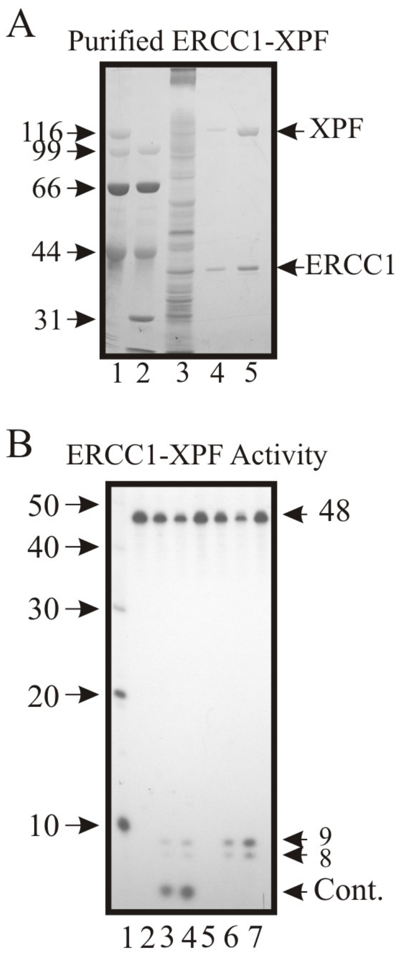

Supplementary Figure S1: A. Overexpression and purification of ERCC1-XPF. Lane 1, high molecular weight protein marker; lane 2, low molecular weight protein marker; lane 3, cell-free extract; lane 4, nickel-NTA agarose pool and lane 5, S-Sepharose pool. **B.** Incision assay using a synthetic 48-mer, with a 22 base loop measuring the nuclease activity of ERCC1-XPF. Lane 1, 10bp marker; lane 2 and lane 3, fractions with contaminating exonuclease activity; lanes 6 and 7, S-sepharose fractions indicating the correct ERCC1-XPF incision products. The incision should produce products between 8-9 bases as indicated.

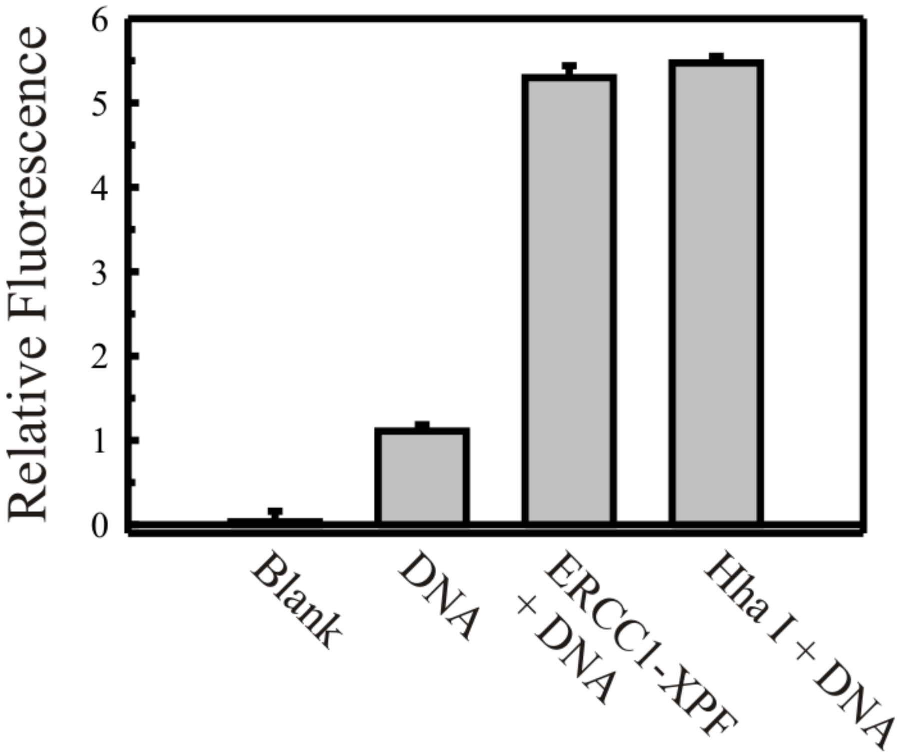

Supplementary Figure S2: Cuvette based fluorescence assay using forked DNA substrate with internal molecular beacon. The relative fluorescence was monitored in a cuvette to assess an increase in fluorescent signal following ERCC1-XPF or HhaI incubation. Blank, buffer alone; DNA, substrate alone without enzyme; ERCC1-XPF plus DNA; HhaI plus DNA.

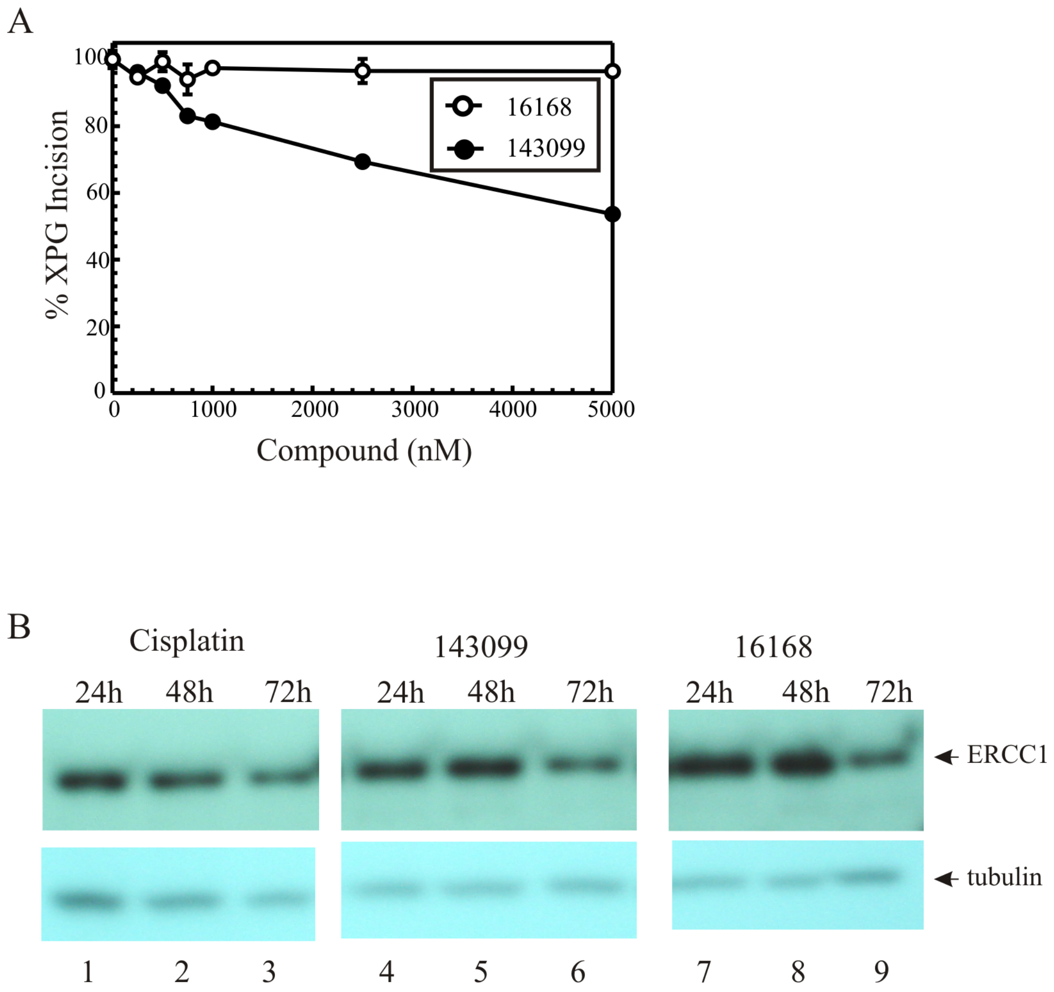

Supplementary Figure S3: A. Analysis of 16168 and 143099 on XPG incision activity. Compounds were titrated and inhibition of XPG incision activity was quantified for 16168 (open circles) or 143099 (filled circles). The results are presented as the mean ± SEM of 3 independent experiments. **B.** Western bots assessing ERCC1 protein stability following cisplatin (IC50 concentration), 143099 (50 µM) and 16168 (50 µM) treatment for 24, 48 and 72 hours.

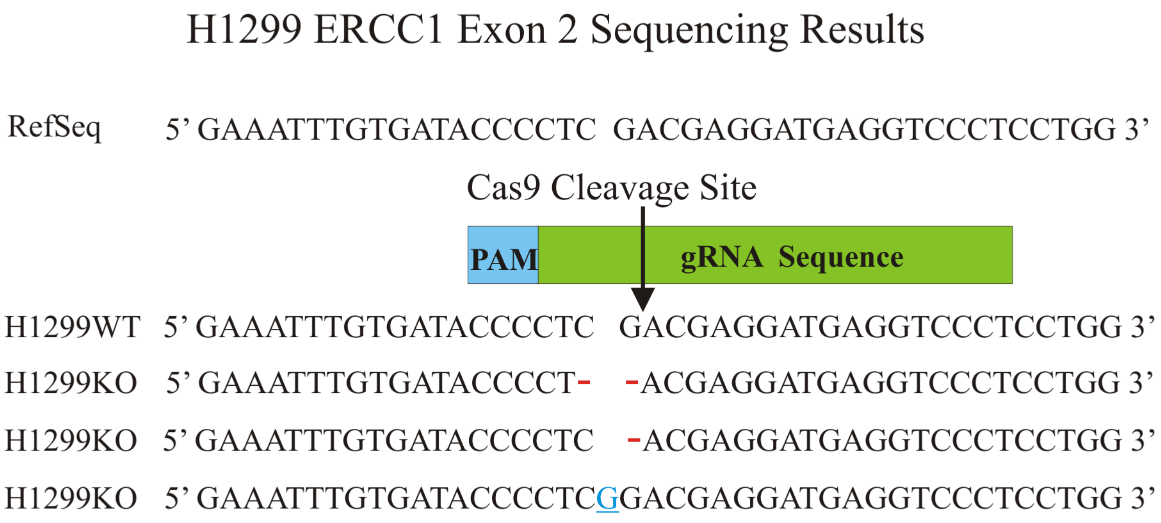

Supplementary Figure S4: Sequencing results for ERCC1 exon 2 near the Cas9 cleavage site. The sequences obtained from H1299 ERCC1 WT cells all matched the RefSeq. Three different edited reads were obtained for H1299 ERCC1 KO cells.

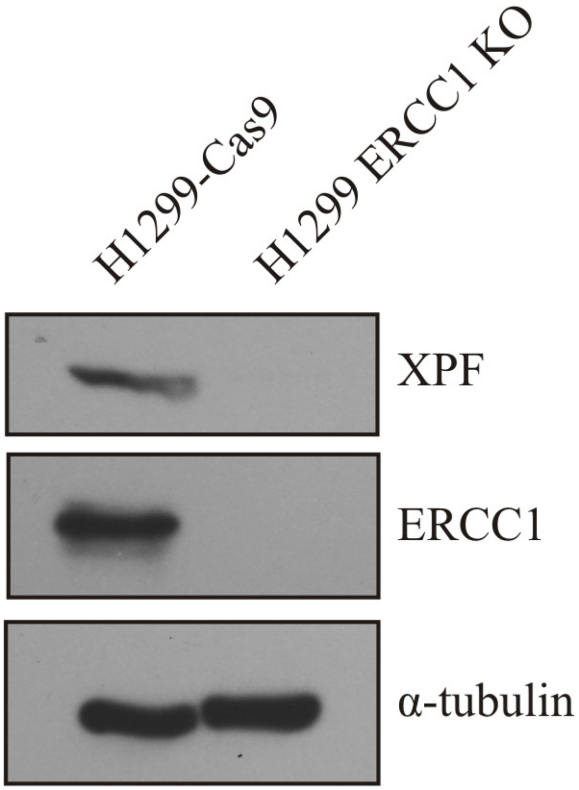

Supplementary Figure S5: Western blot of H1299 ERCC1 WT and ERCC1 KO cells. Blot was probed for XPF (3F2/3 monoclonal antibody), ERCC1 (FL-297 polyclonal antibody), and α-tubulin.

A

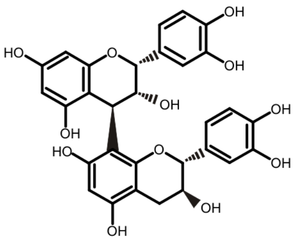

Procyanidin B1
Fl. assay: 900 nM IC50
cell culture: no effect

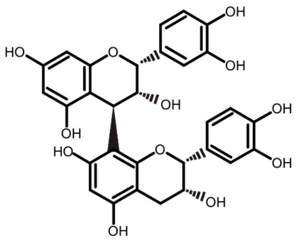

Procyanidin B2
Fl. assay: 500 nM IC50
cell culture: no effect

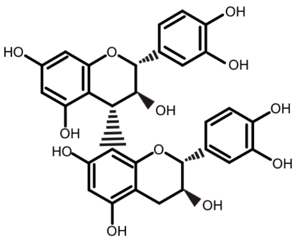

Procyanidin B3
Fl. assay: 250 nM IC50
cell culture: 19.5 µM

B

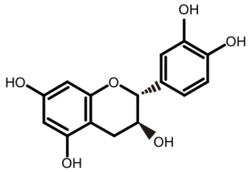

(+)-catechin
Cas #: 154-23-4
Fl. assay: 19 µM IC50
cell culture: no effect

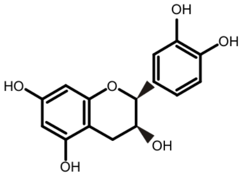

(+)-epicatechin
Cas #: 490-46-0
Fl. assay: >25 µM
cell culture: no effect

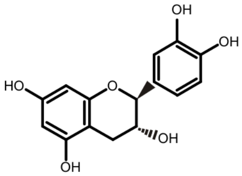

(-)-catechin
Cas #: 18829-79-4
Fl. assay: >25 µM
cell culture: no effect

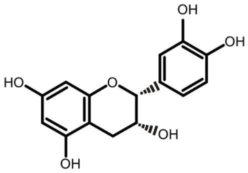

(-)-epicatechin
Cas #: 35323-91-2
Fl. assay: >25 µM
cell culture: no effect

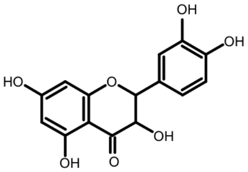

Cas #: 215257-15-1
Fl. assay: >25 µM
cell culture: no effect

C

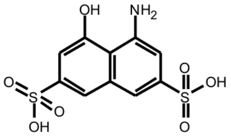

Cas 5460-09-3
Fl. assay: ~3.5 µM IC50
cell culture: no effect

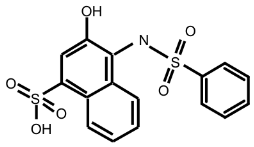

AG-690/36490010
Fl. assay: >25 µM IC50
cell culture: no effect

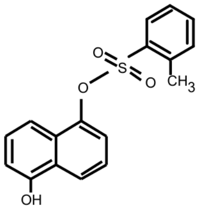

AG-548/43179567
Fl. assay: >25 µM IC50
cell culture: no effect

Supplementary Figure S6: A. Hit 1/NSC143099 structural analogues with differing stereochemistry and assessment in fluorescence assay for ERCC1-XPF inhibition and cell culture experiments to potentiate cisplatin. **B.**, structural analogues of Hit 1 and assessment in incision and cell culture. **C.**, structural analogues of Hit 2/NSC16168 and results in the HTS screen assay and cell culture experiments.

Supplementary Table S1: Off-target sequences for ERCC1 gRNA 5'AGGGACCTCATCCTCGTCGAGGG3'

Gene name	Probability of Off-target editing (0.0-100.0)	Off-target site in coding sequence?	Primers
LIM and SH3 protein 1	0.6	Yes; Exon 7	FW: 5' AAATGCTCAGACCCAGGTGAGC 3'<br>RV: 5' TCAGATGGCCTCCACGTAGTTTG 3'
Leiomodin 2 (cardiac)	0.6	Yes; Exon 1	FW: 5' TTGTTGACCAGCCTGCCACTTG 3'<br>RV: 5' TTTCCACATTCCCCCAGCCTCT 3'
WW and C2 domain containing 1	0.3	Yes; Exon 11	FW: 5' GCTTCACTGACCTCTACTATGACC 3'<br>RV: 5' TTTCCTGGGCGCTGTTACCAA 3'
FAT atypical cadherin 1	0.2	Yes; Exon 4	FW: 5' TACTGTGACAGACAATGGTAGTCC 3'<br>RV: 5' AACCTCTTGGACGAAACCACTCC 3'
Telomeric repeat binding factor 2	0.1	No	
Proteasome 26s subunit ATPase 4	0.1	No	
Mortality factor 4 like 2	0.1	Yes; Exon 1	FW:5' GGGAGTTAGAATGTGTTATTCTCTGATGG 3'<br>RV: 5' AGCTGCTTCTGCCTGGTAACTAA 3'
Nucleoporin 188kDa	0.1	Yes; Exon 10	FW: 5' GTCTCTAGTGAGTTTCAGAACGGG 3'<br>RV: 5' CTGACAAATAAGCCCATCCTGCG 3'
Zinc-finger DHHC-type containing 11	0.1	No	
Zinc-finger protein 677	0.1	No	
Poly-U binding splicing factor 60 kDa	0.0	Yes; Exon 7	FW: 5' CCGTCACCACCATGAAGCACAA 3'<br>RV: 5' AAGAGGCGGTGAGATGGAAAGAC 3'
Tumor protein p73	0.0	Yes; Exon 5	FW: 5' TACCTCTCTGCACCTGACATGG 3'<br>RV: 5' ACCCGTACAGCTGACTGCAG 3'
PSMG3 antisense RNA 1	0.0	No	
A-kinase anchoring protein 1	0.0	No	

Sites of potential off-target editing for the ERCC1 gRNA identified by a genome wide search using the Optimized CRISPR Design Tool. Table 1 contains all identified hits located within gene-coding regions. Column 1 lists the gene names. Column two depicts the algorithm-generated probability off off-target editing for each identified off-target ranging from a score of 0.0-100.0. Column 3 addresses whether the site of potential off-target editing occurs within the mature mRNA. PCR amplification of sites of potential off-target editing in protein coding regions was performed using the primers listed in column 4. After sequencing, there were no genomic edits identified in these potential off-target genes.
